# The role of change agents in achieving quality improvement

**DOI:** 10.1186/1472-6963-14-S2-P138

**Published:** 2014-07-07

**Authors:** Gera Welker, Alice Hobo, Eva Danchell, Jet Van der Weerd, Kees Ahaus

**Affiliations:** 1 Knowledge Center Quality & Safety, University Medical Center Groningen (UMCG), Groningen, The Netherlands; 2 Perioperative Center, UMCG, Groningen, The Netherlands; 3 Program Safety Management System, UMCG, Groningen, The Netherlands; 4 Medical Microbiology, UMCG, Groningen, The Netherlands; 5 Healthwise Center of Expertise on Health Care Management, University of Groningen, Groningen, The Netherlands

## Background

Several UMCG-wide implementation projects improving quality and patient safety have been organized to stimulate the (intrinsic) motivation to work safely in order to bring about the intended change. We deployed ‘change agents’ in various involved groups of professionals and trained them in supporting and stimulating their colleagues to work safely according to the latest evidence based guidelines. We will discuss the effects on implementation success of this strategy, using three examples of improvement projects, namely the implementation of a screening instrument for elderly patients to identify frailty, the Surgical Patient Safety System checklist (SURPASS) in the perioperative process, and the guideline Personal Hygiene.

## Materials and methods

We used e-learning modules, instruction sessions, follow-up sessions, feedback, e-mail and the intranet to inform and instruct the change agents. We measured the effects on improving quality and patient safety by a pre-post evaluation of the degree of implementation, partly by individual completion rates of the checklist and the screening instrument and by observation of personal hygiene indicators. Furthermore, we investigated the influence of leadership and team climate on the effectiveness of deploying change agents [1] using validated questionnaires, and qualitative data analysis of process reports, interviews with change agents and change recipients, and observations.

## Results

The study showed a positive effect of the change agent on the degree of implementation of the various innovations (using SURPASS, using the screening instrument and personal hygiene).

A transformational leadership style of change agents resulted in higher usage rates of change recipients. Climate within the team in which the change agent acts also positively influences the degree of implementation. From the qualitative data we also learned that both the perceived status of the role by change agents themselves and the feedback received on the achieved degree of implementation seem to be affecting the success of a change agent in motivating and stimulating their colleagues to work according to the new guideline.

## Conclusions

Deploying change agents is an effective strategy in encouraging implementation of guidelines. The effect is larger when change agents show transformational leadership and work in a positive team climate. When change agents perceive their added value in implementation success they can motivate their colleagues better.
